# Supplementary material for: Membrane Chromatography-Based Downstream Processing for Cell-Culture Produced Influenza Vaccines
Source: Vaccines (Basel). 2022 Aug 13;10(8):1310. doi: 10.3390/vaccines10081310 (PMC9414887; doi:10.3390/vaccines10081310)
Supplement: Supplementary file 1 [file vaccines-10-01310-s001.zip › vaccines-1806231-supplementary.pdf]

Supplementary Materials

Membrane Chromatography-Based Downstream Processing for Cell-Culture Produced Influenza Vaccines

Zeyu Yang [1,†], Xingge Xu [1,†], Cristina A. T. Silva [1,2], Omar Farnos [1], Alina Venereo-Sanchez [1], Cécile Toussaint [1], Shantoshini Dash [1], Irene González-Domínguez [1,‡], Alice Bernier [1], Olivier Henry [2] and Amine Kamen [1,*]

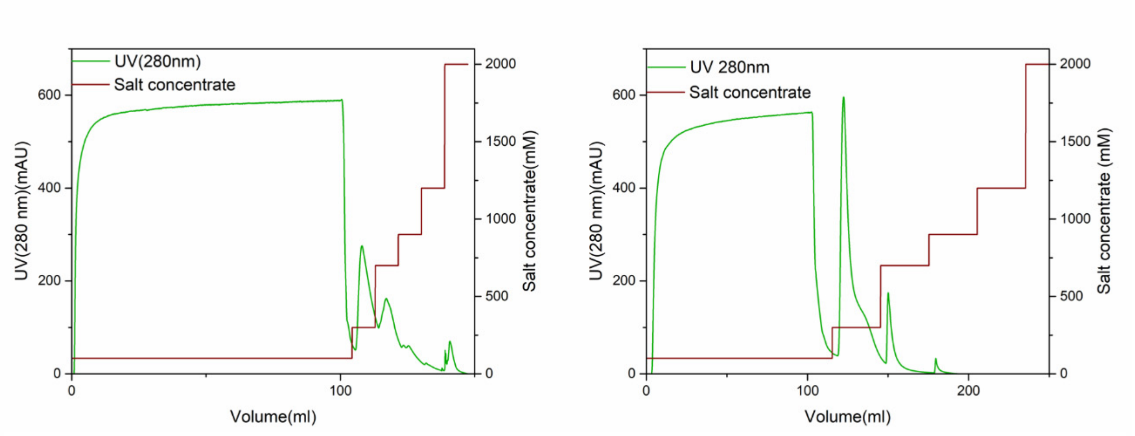

Figure S1. UV and salt concentration curves of Mustang® Q (left) and Sartobind® Q (Right) from AKTA.
